# A Rare Case of Melioidosis in the Bronx

**DOI:** 10.7759/cureus.57277

**Published:** 2024-03-30

**Authors:** Vince Martinez, Jaha Oh, Mukti Gandhi, Walker Brendon, Jamie K Lemon, Addi Feinstein, Alexander Lafortune

**Affiliations:** 1 Department of Internal Medicine, NYC Health and Hospitals/Lincoln, Bronx, USA; 2 Department of Internal Medicine, NYU Langone Hospital - Brooklyn, Brooklyn, USA; 3 Department of Emergency Medicine, NYC Health and Hospitals/Lincoln, Bronx, USA; 4 Department of Pathology and Laboratory Medicine, Northwell Health and Donald and Barbara Zucker School of Medicine at Hofstra/Northwell, Queens, USA; 5 Department of Infectious Diseases, NYC Health and Hospitals/Lincoln, Bronx, USA

**Keywords:** non-endemic region, occupation health, burkholderia pseudomallei, systemic melioidosis, melioidosis

## Abstract

Melioidosis is caused by a gram-negative bacillus *Burkholderia pseudomallei (B. pseudomallei)*, which is found in water and soil in endemic areas. There are indicators that *B. pseudomallei* is increasing in endemic regions and expanding into new locations. It is unclear whether this is because of expanded boundaries or improved detection capabilities. It is even theorized to be endemic in certain parts of the USA. The most common medical risk factor is diabetes mellitus, and it frequently presents as acute pneumonia, and often progresses to bacteremia. It is designated as a tier 1 select biological agent and toxin by the CDC. In this case report, we present a 67-year-old male with multiple comorbidities, who contracted melioidosis while visiting Honduras, as well as the laboratory’s response to the occupational exposure.

## Introduction

Melioidosis, also known as Whitmore’s disease, is an infection caused by a gram-negative soil-dwelling bacterium, *Burkholderia pseudomallei* (*B. pseudomallei*). Infection typically occurs from contact with moist soil or contaminated water, either through direct inoculation or inhalation. It is endemic to tropical and subtropical regions worldwide, particularly in Southeast Asia and Australia. Certain environmental conditions can increase the risk of exposure, primarily high levels of humidity and precipitation [1]. Adults with preexisting comorbidities, such as diabetes mellitus, alcohol use disorder, chronic renal disease, and chronic lung conditions, are more likely to be affected by the disease [2-4]. Manifestations of the disease are variable and can range from a nonspecific viral syndrome to pneumonia, renal failure, and bacteremia with hematological seeding, to distant sites in the form of visceral abscesses. Chronic melioidosis can develop if symptoms last longer than two months, and it is seen in about 11% of cases [5]. Diagnosis is usually made based on the clinical picture, epidemiology, and bacterial culture. Culture-based bacterial identification has been considered the diagnostic gold standard [6]. Infection by exposure through aerosols or cutaneous inoculation has occurred in laboratory workers, which highlights the importance of safe laboratory practices [7]. Our case demonstrates a great contribution from laboratory workers as it is rare to see a case of melioidosis in the USA, which likely occurred from a recent travel to an endemic region.

## Case presentation

A 67-year-old male, an immigrant from Honduras residing in New York City for the past 24 years, with a past medical history of uncontrolled insulin-dependent diabetes mellitus, hypertension, and an automatic implantable cardioverter defibrillator (AICD) for unspecified cardiac disease, was brought to the emergency department by his family because of a five-day history of altered mental status. Preceding his presentation, he spent four months in a coastal area in Honduras at a time when the precipitation was uncharacteristically heavy. Toward the end of his stay, he was hospitalized for a urinary tract infection (UTI) associated with an acute kidney injury (AKI) requiring intravenous (IV) antibiotics and rehydration. He was transitioned to oral antibiotics and was discharged; however, he did not complete the antibiotic course. He was readmitted for acute altered mental status with frequent falls and was found to have persistence of UTI, as well as worsening AKI. He was treated with IV antibiotics and hydration for three days without improvement in his mental status, and then he was discharged home with antibiotics. He returned to the USA two days after the discharge. However, he was still disoriented and becoming drowsier, so the family brought the patient to the hospital the day after returning to the USA. On presentation, he was hemodynamically stable with mild hypothermia and altered mental status, triggering a sepsis screen. Initial physical examination was remarkable for mild suprapubic tenderness to palpation, somnolence, and disorientation. Initial laboratory tests (Table 1) revealed leukocytosis, thrombocytopenia, hyperglycemia, acute renal failure, mild transaminitis, and significant metabolic derangements, including high anion gap metabolic acidosis secondary to uremia, lactatemia, and ketosis. Other labs were remarkable for erythrocyte sedimentation rate (ESR) of 120 mm/hour and procalcitonin of 9.09 ng/mL. His urinalysis was not suggestive of a UTI.

**Table 1:** Initial laboratory tests Complete blood count, comprehensive metabolic panel, and urine analysis. ALT: alanine aminotransferase; AST: aspartate aminotransferase; dL: deciliter; ESR: erythrocyte sedimentation rate; fL: femtoliter; g: grams; HPF: high-power field; mcL: Microliter; MCV: mean corpuscular volume; Mg: milligrams; mL: millileters; mm: millimeters; Mmol: millimole; ng: nanograms per milliliter; pg: picograms; pH: potential of hydrogen; PLT: platelet count; RBC: red blood cell count; U/L: units per liter; WBC: white blood cell count.

	Reference Value	Reference Units	Patient’s Value
WBC	4.8–10.8	x 10^3^/mcL	13.5
RBC	4.7–6.1	x 10^6^/mcL	4.79
Hemoglobin	14.0–18.0	g/dL	12.9
Hematocrit	42–52	%	36.2
MCV	80–99	fL	75.6
PLT	150–450	x 10^3^/mcL	100
Neutrophil %	44–70	%	92.1
Lymphocyte %	20–45	%	3.9
Sodium	136–145	Mmol/L	129
Potassium	3.5–5.1	Mmol/L	4.9
Chloride	98–107	Mmol/L	91
Carbon Dioxide	22–29	Mmol/L	16
Anion Gap	5–17	Mmol/L	22
Blood Urea Nitrogen	6–23	Mg/dL	114
Creatinine	0.7–1.2	Mg/dL	8.15
Glucose	74–109	Mg/dL	344
Calcium	8.4–10.5	Mg/dL	8.8
Albumin	3.5–5.2	g/dL	2.3
Alkaline Phosphatase	40–130	U/L	424
ALT	<=41	U/L	51
AST	<=40	U/L	55
Pro B-Natriuretic Peptide	<=124	pg/mL	7,730
Troponin T	<=0.01	ng/mL	<0.01
Lipase	13–60	U/L	46
Lactic Acid	0.5–2.2	Mmol/L	1.8
Creatine Kinase	20–200	U/L	294
ESR	0–15	mm/hr	120
Urinalysis			
pH	5–8		5.0
Color			Yellow, cloudy
Glucose	Negative		250
Protein	Negative		100
Ketones	Negative		trace
Specific Gravity	1.01–1.025		1.015
RBC	0–4	/HPF	0-6
WBC	0–5	/HPF	4-10
Bacteria	Negative		Negative
Leukocyte Esterase	Negative		Negative
Nitrite	Negative		Negative
Squamous Cells	0–5	/HPF	0-5

**Figure 1:**
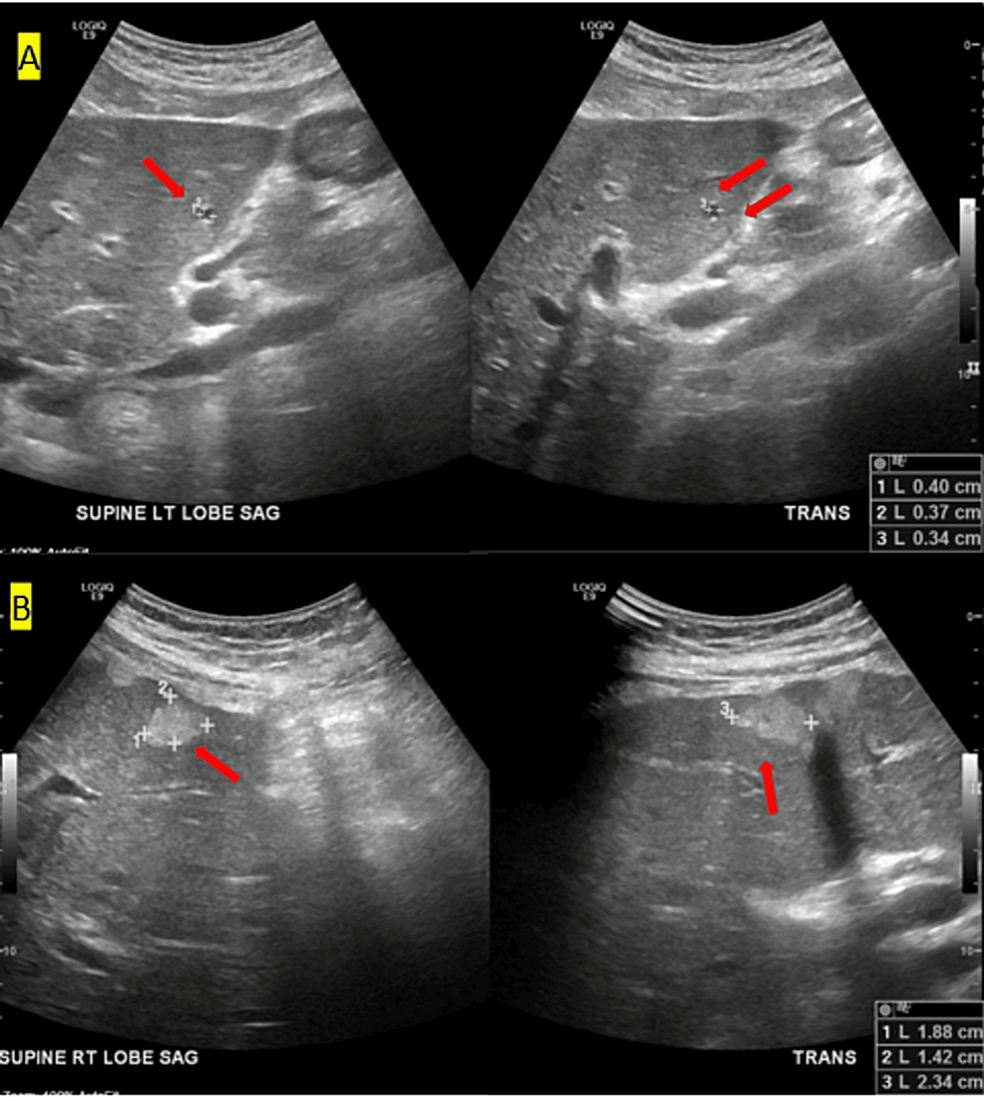
Initial liver ultrasound Figure A: Hepatomegaly with sub-centimeter hepatic cysts on the left lobe; Figure B: Hyperechoic lesions consistent with hemangiomas on the right lobe.

Chest radiograph (CXR) was unrevealing acute cardiopulmonary disease. A non-contrast head CT was nonsuggestive of acute intracranial pathologies. Initial liver ultrasound (US) showed hepatomegaly with subcentimeter hepatic cysts on the left lobe (Figure 1A) and hyperechoic lesions consistent with hemangiomas on the right lobe (Figure 1B). Given the altered mental status with systemic inflammatory response, empiric antibiotic treatment for meningitis was commenced. However, a lumbar puncture was not pursued given the patient’s thrombocytopenia and uremia. The patient was admitted to the inpatient medical service.

During his inpatient stay, the patient’s mental status and renal dysfunction persisted, while his leukocytosis continued to an uptrend. The preliminary blood cultures resulted in gram-negative bacilli. Infectious disease was consulted, and the antibiotics were escalated to meropenem 1 g daily based on renal function. The patient’s specimen was sent to Northwell laboratories for culture. While they were dealing with a tier 1 select agent, they discovered the events described below.

**Figure 2:**
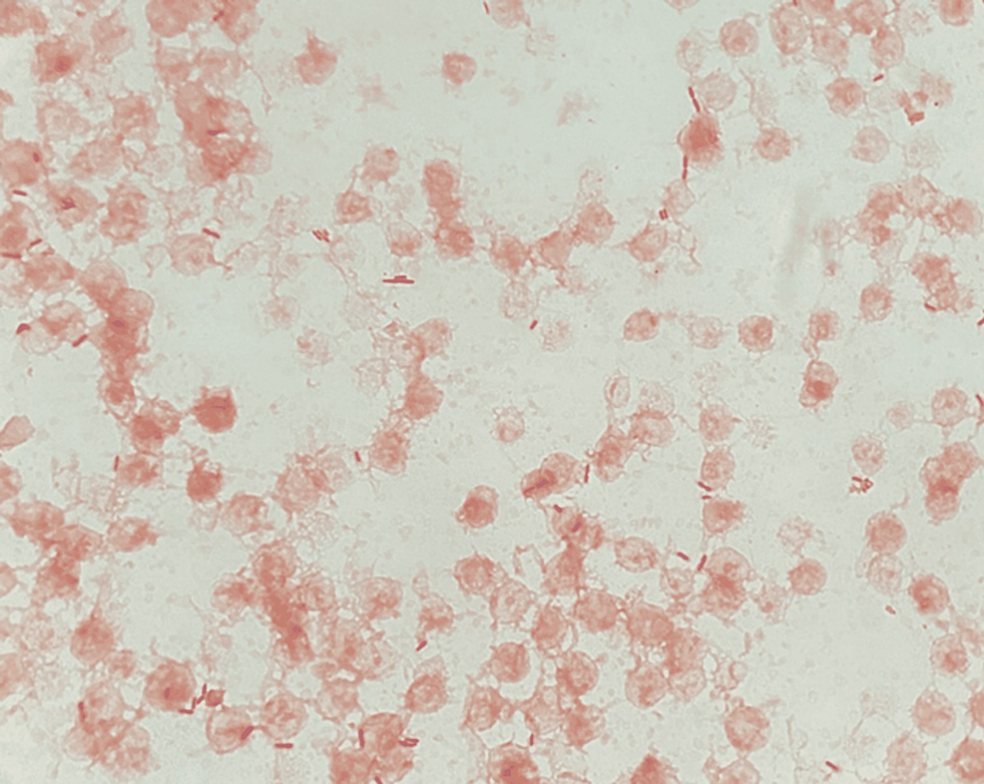
Gram-staining of the aerobic blood culture bottle Demonstrated the presence of slender, gram-negative rods. No bipolar staining was observed (1,000x magnification).

After 22.08 hours of incubation, the aerobic bottle flagged positive, and staining demonstrated the presence of gram-negative rods (Figure 2). The positive bottle was subcultured and incubated at 35°C for 12 hours in a total laboratory automation system. The growth of a non-lactose-fermenting organism with small gray colonies on sheep blood agar (SBA) was observed and worked up for identification by matrix-assisted laser desorption/ionization-time of flight mass spectrometry (MALDI-ToF MS). Initial MALDI-ToF MS results yielded no identification on an FDA-cleared in vitro diagnostic (IVD) database and identification of *Burkholderia thailandensis* (score 2.01) on the research use only (RUO) database. Antimicrobial susceptibility testing (AST) was set up using an automated MIC method (MicroScan NM56 Panel, Beckman Coulter, Brea, CA) and incubated at 35°C for 18 hours. The following day, a second attempt was made at MALDI-ToF MS identification. Results were similar to the previous day; however, on this attempt, the technologist reviewing the MALDI-ToF results recognized *B. thailandensis* as a common misidentification for *B. pseudomallei* and initiated the appropriate rule-out procedure. The isolate was oxidase-positive and indole-negative and grew on polymyxin B-containing agar. Therefore, *B. pseudomallei* could not be ruled out. The New York City Public Health Lab (NYC PHL) Biothreat laboratory was contacted, and the isolate was referred for further identification. The isolate was presumptively identified as *B. pseudomallei* by polymerase chain reaction (PCR) at NYC PHL, and this identification was confirmed by definitive testing at the CDC.

Multiple factors contributed to the mishandling of this isolate in the Northwell laboratory, resulting in high-risk exposures of four laboratory staff members. The first was the rapid growth of this isolate in blood culture bottles. Additional safety precautions for handling potential biothreat agents were in place but required growth at more than 48 hours of incubation for these precautions to be implemented. The second was the initial failure to recognize the MALDI-ToF identification of *B. thailandensis *as a potential misidentification of *B. pseudomallei*. The third was the practice of setting up AST panels on the open bench before having a definitive organism identification, which has now been discontinued for all blood culture isolates. In addition to rapid growth in blood culture bottles and subculture plates, this isolate of *B. pseudomallei* lacked several of the characteristics associated with this species, including bipolar gram-staining, wrinkled colonies at more than 48 hours of incubation, and distinctive “musty” or “earthy” odor. All exposed employees were offered postexposure prophylaxis and serologic monitoring, with no seroconversions observed.

**Figure 3:**
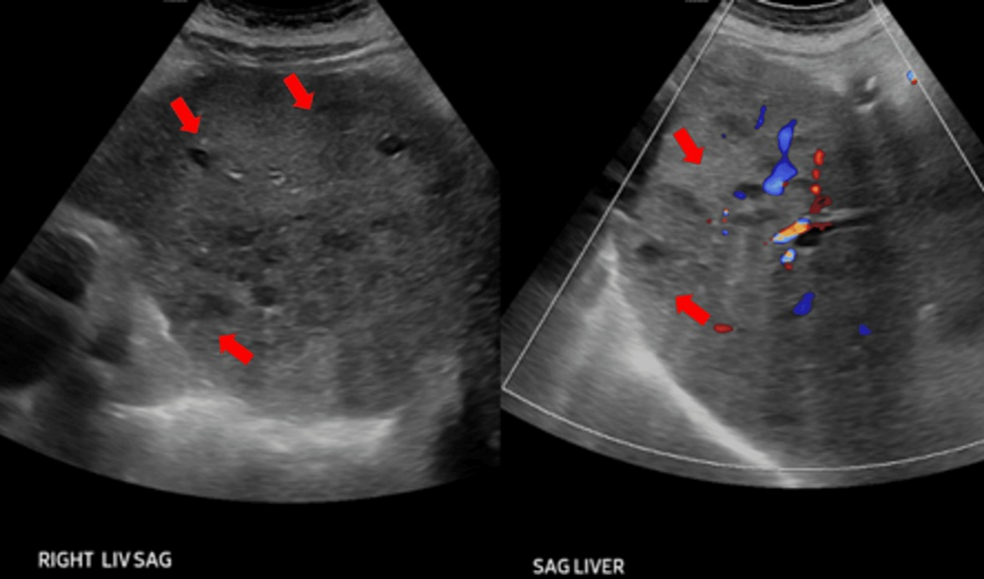
Repeated liver US Innumerable, small, and hypoechoic lesions scattered throughout the liver, suspicious for abscesses.

**Figure 4:**
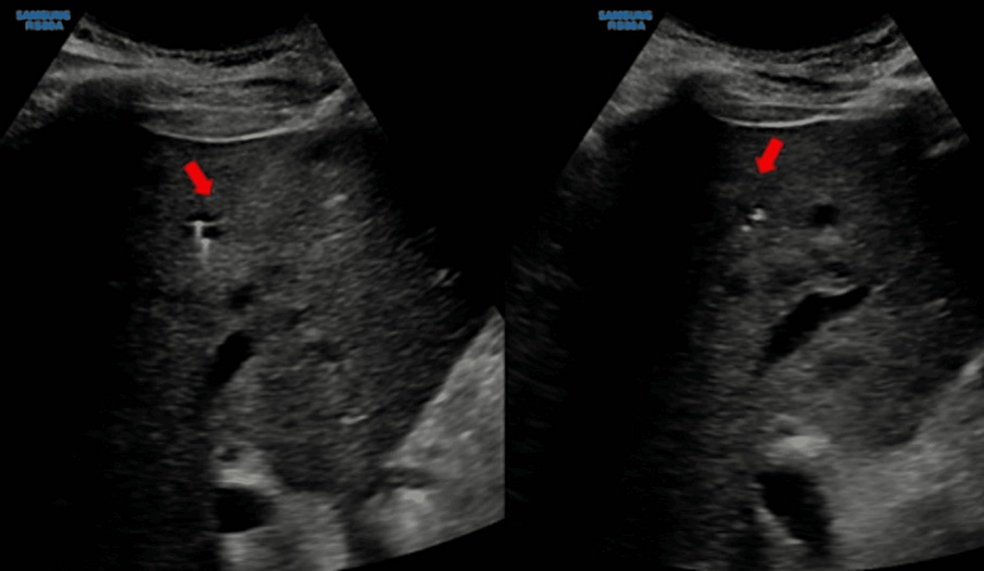
Percutaneous aspiration of the tiny liver abscess within the right lobe of the liver Culture revealed *Burkholderia pseudomallei*.

Additionally, CT chest, abdomen, and pelvis without contrast were pursued to investigate for infective foci and revealed a 5 cm liver mass with innumerable smaller lesions throughout the liver, a nonspecific 2 cm hypodense lesion at the inferior pole of the spleen, small pleural effusions with subjacent atelectasis, and an enlarged prostate with mildly thickened bladder wall, likely chronic hypertrophy. Tumor markers for liver lesions, including alpha-fetoprotein (AFP), carcinoembryonic antigen (CEA), and carbohydrate antigen 19-9 (CA19-9), were nonremarkable. Repeated liver ultrasound described multiple tiny cystic-like structures and regions of increased echogenicity, consistent with abscesses rather than cysts (Figure 3). IR-guided biopsy and aspiration of the cystic structures were pursued (Figure 4). Cytology and culture revealed *B. pseudomallei*. Transthoracic echocardiogram did not show valvular or AICD lead vegetations. A renally adjusted high dose of meropenem was continued for an extended initial intensive phase of treatment, and Bactrim (sulfamethoxazole and trimethoprim) was added as an adjunctive therapy given encephalopathy and hepatic involvement. Following appropriate treatments with antibiotics and aspiration of live abscesses, the patient’s mental status returned to baseline, leukocytosis resolved, and renal function gradually improved. Serial blood cultures became seronegative on day seven of appropriate antibiotics. The patient was then discharged to a subacute rehabilitation to complete a six to eight week of an intensive-phase antibiotics course with both meropenem and Bactrim.

**Figure 5:**
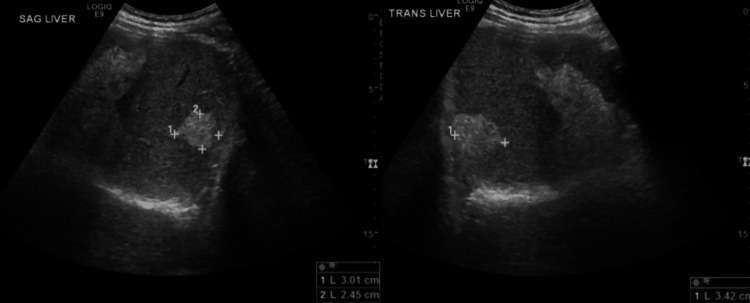
Serial liver ultrasound after discharge Multiple hyper-echogenicities up to 5 cm throughout the liver.

**Figure 6:**
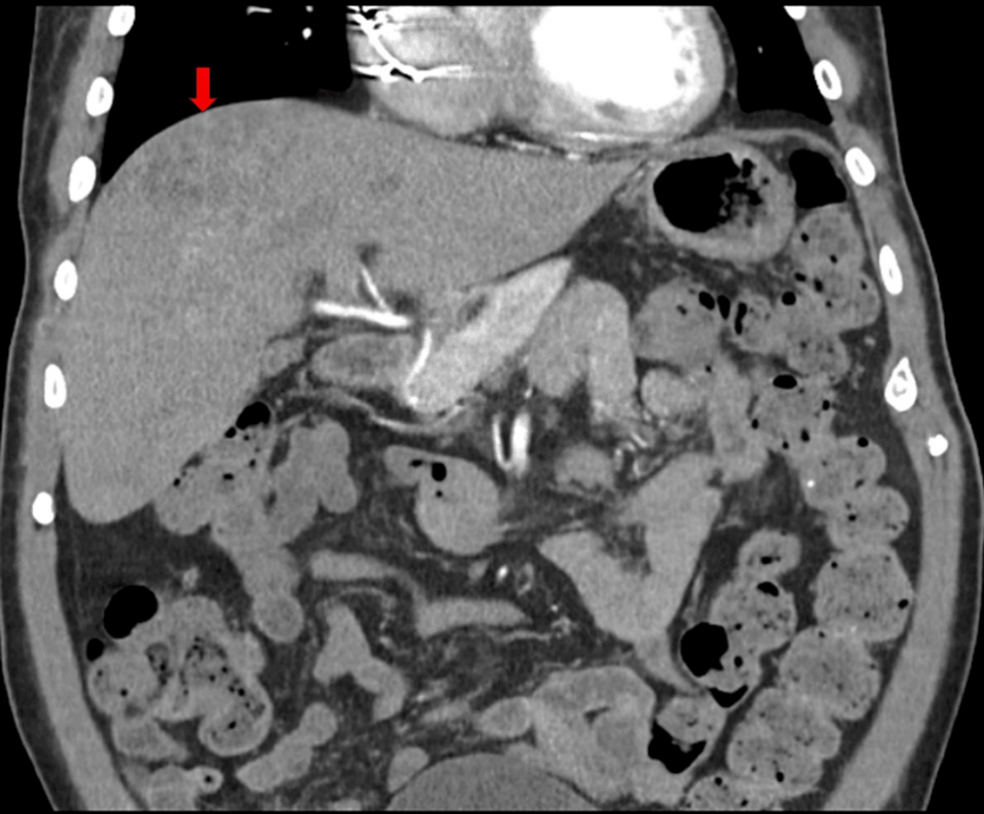
Contrast-enhanced liver CT Wedge-shaped heterogeneous areas of low attenuation in segment VIII of the liver, likely the sequela of prior infection but no evidence of abscess.

The patient followed up in the infectious disease clinic at week six post discharge and then every three to four weeks afterward. Serial liver US revealed stable multiple echogenic structures throughout the liver (Figure 5). Contrast-enhanced liver CT revealed wedge-shaped heterogeneous areas of low attenuation in segment VIII of the liver (Figure 6), likely the sequela of prior infection but no evidence of abscess. Considering these CT findings and clinical improvement, the decision was made to discontinue meropenem after 76 days of treatment. Bactrim was changed to doxycycline because of a reduction of renal function and is to be continued for three months for the "eradication phase"/suppression, which is suboptimal but appropriate given the adverse effects of Bactrim. 

## Discussion

History and microbiology

Melioidosis is an acute infection caused by the bacterium *B. pseudomallei*. It was first recognized by a British medical officer, Dr. Alfred Whitmore, in a 1913 article recounting his investigations on two of his patients who died from septicemia in 1911 while he was stationed in Burma [8,9]. He soon realized that they were dealing with an unfamiliar infectious disease [8,9]. After performing several microbiological tests to describe the organism, he proposed the name *Bacillus pseudomallei* [8,9]. This was then heralded as the “new disease of the tropics” by authors Stanton et al. in 1921 who proposed the name melioidosis, from the Greek word "melis," which pertains to the variety of conditions resembling glanders [10,11]. It underwent a few name changes. At one time, it was under the genus *Pseudomonas*, until *Burkholderia *was separated into its own genus based on the heterogeneity of ribosomal RNA (rRNA) [6].

It exists in nature as a saprophyte and may make its way to humans through broken skin, inhalation, or ingestion [9]. It is a disease originating from hot and humid regions within tropical countries and is most prevalent in Northern Territory, Australia, and northeast Thailand [5,12]. It is also found in India, Bangladesh, Indonesia, and other Southeast Asian countries, with sporadic cases reported across the globe, such as in Brazil, Africa, and even in the USA [5]. Besides being present in soil and surface water, outbreaks have occurred because of contaminated water supplies and nosocomial dissemination [5]. Certain environmental conditions (e.g., tropical storms) and certain occupations (e.g., rice farming) also increase the risk of exposure to this organism [5]. In recent decades, the geographic range has expanded considerably, and it is now suspected to be endemic in parts of Central and South America, the Caribbean, Mexico, and potentially certain parts of the USA [13]. Most case reports in the USA are related to international travel to endemic areas, whereas some cases were related to the importation of products (e.g., aromatherapy spray, tropical fish) from endemic areas [13]. There is a case report in rural Texas, the infection of which is theorized to be acquired from the environment [14]. However, after a follow-up investigation, endemicity was not established, and the source remains unknown [14].

In our patient, he travels back to his home country in Honduras almost every year and experienced heavy rains on his last trip. Although he denied water-related activities, environmental exposure likely played a role in his subsequent infection, especially as tropical storms have been known to cause outbreaks [1]. 

Clinical diagnosis, disease, and treatment

The most common medical risk factor is diabetes mellitus [5]. Although most exposures may have a subclinical manifestation, some will develop into either an acute, chronic, or latent disease [5]. The incubation period varies from one to 21 days, and it is nine days on average [5]. Most cases are acute infections, usually presenting with pneumonia [5]. As most clinical presentations are nonspecific, this disease should be considered in any patient who has the risk factors and who has a travel history from endemic areas [5].

In this patient, who recently went to South America with uncontrolled diabetes and possible aquatic exposure, it was the perfect storm for melioidosis to occur. Although the patient did not have pulmonary disease, mild transaminitis in his initial blood workup hinted at a liver pathology, which was evident in the ultrasonography and computed tomography. Similar to any case of bacteremia, early diagnosis and antibiotic administration are crucial. In the initial intensive therapy, ceftazidime and meropenem are the preferred agents for 10-14 days, while trimethoprim-sulfamethoxazole is recommended for the subsequent eradication therapy, and given for up to three to six months [5]. When the patient was discharged to the nursing facility, meropenem was continued, together with Bactrim. Adjuvant therapy to be considered in treatment includes the granulocyte colony-stimulating factor used to counteract the functional neutrophil defects vital in the pathogenesis of melioidosis [5]. Studies have also shown possible roles for interleukin-1 beta-blocking agents, interleukin-7, and anti-PD1 agents [5].

Laboratory safety/occupational exposure

Airborne transmission to humans was not demonstrated until a case report in 1968 described occupational exposure when a centrifuge containing the organism developed a leak and sprayed specimens into the air, walls, and work area of a bacteriologist [15]. To date, there has been only one other case of laboratory-acquired melioidosis, but there have been numerous occupational exposures, most of which did not convert into clinical disease [6]. The species under the genus *Burkholderia *are relatively easy to aerosolize when manipulated in the laboratory [16]; thus, this represents the greatest biohazard [7]. Workers in diagnostic laboratories may be unwittingly exposed to this organism before its identity is established [7]; thus, good laboratory practices are of utmost importance. Recognition of this organism in the clinical laboratory is further challenged by the fact that commercial identification platforms, including MALDI-ToF MS, commonly misidentify *B. pseudomallei* as other *Burkholderia *species, particularly *B. thailandensis*. Such identifications should prompt the lab to initiate the Association of Public Health Laboratories/Laboratory Response Network (APHL/LRN) Rule-Out or Refer testing algorithm to ensure that any suspicious isolates are definitively identified. *B. pseudomallei* is designated as a tier 1 select biological agent by the CDC, meaning that it was determined to be at high risk for deliberate misuse, with significant potential for mass casualties or devastating effects on the economy, critical infrastructure, or public confidence [7]. Thus, researchers and facilities handling it must be registered, inspected, cleared, and approved by the proper federal agencies before beginning their research [7]. The guidelines suggest handling this organism in a biosafety level 3 facility within a class II biological safety cabinet [17].

## Conclusions

Meliodosis is caused by *B. pseudomallei*, a gram-negative bacterium, endemic to Australia and Southeast Asian countries, and designated as a tier 1 select biological agent and toxin by the CDC, given its high risk for deliberate misuse with significant potential for mass casualties or devastating effects to the economy, critical infrastructure, or public confidence. Therefore, accurate and timely diagnosis is critical in properly managing this condition. We present a case that challenges the geographical distribution, highlights portions of the natural history of the disease, and discusses a laboratory's response and corrective actions to occupational exposure.
